# *Tettigettalna josei* (Boulard, 1982) (Hemiptera: Cicadoidea): first record in Spain, with notes on the distribution, genetic variation and behaviour of the species

**DOI:** 10.3897/BDJ.2.e1045

**Published:** 2014-05-09

**Authors:** Paula C. Simões, Vera L. Nunes, Raquel Mendes, Sofia G. Seabra, Octávio S. Paulo, José A. Quartau

**Affiliations:** † Faculdade de Ciências da Universidade de Lisboa, Departamento de Biologia Animal e Centro de Biologia Ambiental, Computational Biology and Population Genomics Group, Edifício C2, Piso 3, Campo Grande, 1749-016, Lisbon, Portugal

**Keywords:** Cicada, distribution, first record, Spain

## Abstract

The small cicada *Tettigettalna josei* (Boulard, 1982) was until recently only known from southern Portugal and was considered endemic to this country. Fieldwork in 2013 led to the first record of the species in Spain, expanding its known eastern range to Andalusia. The northern limits remain poorly defined but it appears that the distribution of *Tettigettalna josei* is restricted to the south Atlantic coastline in the Iberian Peninsula, with the highest densities found in Algarve. Some notes on behaviour and genetic variation of *Tettigettalna josei* are also given.

## Introduction

Cicadas are well known for the acoustic signals produced by males during the mating season to attract females. Male calling songs are species-specific and easily distinguished by trained human ears. The acoustic signals are taxonomically valuable, being effective to distinguish between close cicada species and are especially useful to identify sibling species when they occur in the same geographical area (Boulard 1982).

The biodiversity of cicadas (Hemiptera: Cicadoidea) worldwide is remarkable but many taxa remain poorly known or even undescribed. Recent work in the Iberian Peninsula led to the revision and description of nine species under the genus *Tettigettalna* (Puissant and Sueur 2010). All species comprising the genus are small-sized (body length < 25 mm) and seven species are considered to be endemic to southern Iberia (Puissant and Sueur 2010, Simões et al. 2013, Sueur et al. 2004). The distribution boundaries of these species remain poorly defined because they are based on sporadic records and extensive field surveys have yet to be undertaken. Three *Tettigettalna* species (*Tettigettalna mariae*, *Tettigettalna josei* and *Tettigettalna estrellae*) were described from Portugal and were thought to be endemic to this country, although their distributions in other parts of the Iberian Peninsula had never been investigated. Recent field surveys showed that the distribution of *Tettigettalna mariae* actually extends to the neighbouring country, Spain, and this species is now considered as an Iberian endemism (Simões et al. 2013). As an outcome from intensive fieldwork for species of genus *Tettigettalna* in both southern Portugal and Spain during the summers of 2011-2013, we report here an update on the distribution range of *Tettigettalna josei*, with the discovery of its presence in Andalusia in the summer of 2013.

## Materials and methods

Several field surveys were conducted in the southern Iberian Peninsula (regions of Algarve and Andalusia) from the end of June until mid-August during the summers of 2011–2013. The fieldwork took place from 10:00 am to 07:00 pm with sunny weather and with temperatures ranging from 24 to 39 °C degrees. Initial searches in 2011 were conducted by driving a car at low speed to allow the detection of the calling males. In 2012 and 2013, searches were targeted to areas of potentially suitable habitats. Geographical coordinates were determined with a GPS (Garmin, Oregon series 550t) for each site where male songs of *Tettigettalna josei* were heard or where specimens were collected.

**Figure 1.**
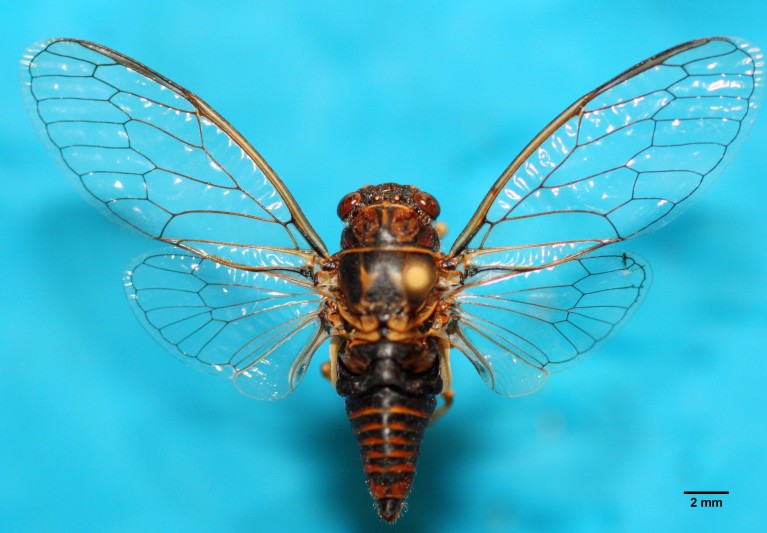
Male of *Tettigettalna josei* collected in Cartaya (ID 3577, Huelva, Andalusia) in July 2013.

Species were located through their calling song and their songs recorded in the field, followed by capture with a sweeping net. Acoustic signals were recorded using a Marantz PMD 661 Portable SD recorder (20 Hz – 24 kHz) connected to a Telinga Pro 7 Dat-mic microphone (Twin Science) following the procedures given in Quartau et al. (1999). Captured specimens were conserved dry (Fig. 1) at the general data bank on insect data at the Department of Animal Biology in the Faculty of Sciences, University of Lisbon (FCUL). A front leg was removed from each specimen and preserved in absolute ethanol for DNA isolation.

Time and frequency analysis of sound recordings from males were conducted with software Avisoft SASLab Pro (Specht 2012) as in previous analyses (e.g. Quartau et al. 1999, Simões et al. 2000). Acoustic recordings were analysed with a sampling rate of 44.1 kHz and a resolution of 16 bits. Spectra were computed using FFT with a resolution of 512 points and a Hamming Window. For each male, recordings of about one minute were analysed. Song terminology follows that of Gogala and Trilar (1999), Gogala and Trilar (2000).

Seven morphologic measures were taken from collected males in Spain as in Boulard (1982), namely, total length (TL), body length (BL), wingspan (WS), right hindwing length (aWL), head width (HW), mesonotum width (MW) and width at the level of the auditory capsules (ACD).

Whole-genome DNA was isolated with the E.Z.N.A.® Tissue DNA Isolation kit (Omega Bio-Tek). Sequences of 581 base pairs from the 5’ region of the cytochrome *c* oxidase I (COI) mitochondrial gene were obtained from 15 males of *Tettigettalna josei*. Sequences were obtained as described in Nunes et al. (2014) and deposited in GenBank (accession numbers KF977491–KF977505). A minimum spanning network was constructed with the median-joining method (Bandelt et al. 1999) in NETWORK 4.6.1.1 (http://www.fluxus-engineering.com). The input file was converted from FASTA to NEXUS format with CONCATENATOR 1.1.0 (Pina-Martins and Paulo 2008, http://cobig2.com/software).

## Taxon treatments

### Tettigettalna josei

(Boulard, 1982)

#### Materials

- **Type status:** Other material. **Occurrence:** recordedBy: Bruno Novais; individualCount: 1; sex: male; **Location:** country: Portugal; stateProvince: Algarve; verbatimLocality: Budens; verbatimLatitude: 37°04'45.2"N; verbatimLongitude: 8°50'11.6"W; **Event:** samplingProtocol: Sweep net capture; eventDate: 2011-07-27; **Record Level:** collectionID: Tjo119; institutionCode: FCUL; collectionCode: Entomology_PCS
- **Type status:** Other material. **Occurrence:** recordedBy: Bruno Novais; individualCount: 1; sex: male; **Location:** country: Portugal; stateProvince: Algarve; verbatimLocality: Budens; verbatimLatitude: 37°04'22.9"N; verbatimLongitude: 8°48'43.9"W; **Event:** samplingProtocol: Sweep net capture; eventDate: 2011-07-27; **Record Level:** collectionID: Tjo122; institutionCode: FCUL; collectionCode: Entomology_PCS
- **Type status:** Other material. **Occurrence:** recordedBy: Bruno Novais; individualCount: 1; sex: male; **Location:** country: Portugal; stateProvince: Algarve; verbatimLocality: Porches; verbatimLatitude: 37°08'09.4"N; verbatimLongitude: 8°23'04.2"W; **Event:** samplingProtocol: Sweep net capture; Acoustic recording; eventDate: 2011-07-26; **Record Level:** collectionID: Tjo113; institutionCode: FCUL; collectionCode: Entomology_PCS
- **Type status:** Other material. **Occurrence:** recordedBy: Bruno Novais; individualCount: 1; sex: male; **Location:** country: Portugal; stateProvince: Algarve; verbatimLocality: Vale Judeu; verbatimLatitude: 37°07'39.8"N; verbatimLongitude: 8°05'36.1"W; **Event:** samplingProtocol: Sweep net capture; Acoustic recording; eventDate: 2011-07-12; **Record Level:** collectionID: Tjo66; institutionCode: FCUL; collectionCode: Entomology_PCS
- **Type status:** Other material. **Occurrence:** recordedBy: Vera Nunes; individualCount: 1; sex: male; **Location:** country: Portugal; stateProvince: Algarve; verbatimLocality: Quinta do Lago; verbatimLatitude: 37°03'35.2"N; verbatimLongitude: 8°01'16.3"W; **Event:** samplingProtocol: Sweep net capture; Acoustic recording; eventDate: 2012-08-01; **Record Level:** collectionID: Tjo309; institutionCode: FCUL; collectionCode: Entomology_PCS
- **Type status:** Other material. **Occurrence:** recordedBy: Vera Nunes; individualCount: 1; sex: male; **Location:** country: Portugal; stateProvince: Algarve; verbatimLocality: Quinta do Lago; verbatimLatitude: 37°03'35.2"N; verbatimLongitude: 8°01'16.3"W; **Event:** samplingProtocol: Sweep net capture; Acoustic recording; eventDate: 2012-08-08; **Record Level:** collectionID: Tjo355; institutionCode: FCUL; collectionCode: Entomology_PCS
- **Type status:** Other material. **Occurrence:** recordedBy: Vera Nunes; individualCount: 1; sex: male; **Location:** country: Portugal; stateProvince: Algarve; verbatimLocality: Quinta do Lago; verbatimLatitude: 37°03'35.2"N; verbatimLongitude: 8°01'16.3"W; **Event:** samplingProtocol: Sweep net capture; Acoustic recording; eventDate: 2012-08-09; **Record Level:** collectionID: Tjo362; institutionCode: FCUL; collectionCode: Entomology_PCS
- **Type status:** Other material. **Occurrence:** recordedBy: Bruno Novais; individualCount: 1; sex: male; **Location:** country: Portugal; stateProvince: Algarve; verbatimLocality: S. Brás de Alportel; verbatimLatitude: 37°08'14.8"N; verbatimLongitude: 7°50'52.4"W; **Event:** samplingProtocol: Sweep net capture; Acoustic recording; eventDate: 2011-08-04; **Record Level:** collectionID: Tjo145; institutionCode: FCUL; collectionCode: Entomology_PCS
- **Type status:** Other material. **Occurrence:** recordedBy: Vera Nunes; individualCount: 1; sex: male; **Location:** country: Portugal; stateProvince: Algarve; verbatimLocality: Moncarapacho; verbatimLatitude: 37°04'41.3"N; verbatimLongitude: 7°49'16.6"W; **Event:** samplingProtocol: Sweep net capture; Acoustic recording; eventDate: 2011-08-03; **Record Level:** collectionID: Tjo141; institutionCode: FCUL; collectionCode: Entomology_PCS
- **Type status:** Other material. **Occurrence:** recordedBy: Bruno Novais; individualCount: 1; sex: male; **Location:** country: Portugal; stateProvince: Algarve; verbatimLocality: Moncarapacho; verbatimLatitude: 37°04'41.3"N; verbatimLongitude: 7°49'16.6"W; **Event:** samplingProtocol: Sweep net capture; Acoustic recording; eventDate: 2011-08-10; **Record Level:** collectionID: Tjo154; institutionCode: FCUL; collectionCode: Entomology_PCS
- **Type status:** Other material. **Occurrence:** recordedBy: Bruno Novais; individualCount: 1; sex: male; **Location:** country: Portugal; stateProvince: Algarve; verbatimLocality: Tavira; verbatimLatitude: 37°08'02.0"N; verbatimLongitude: 7°38'04.2"W; **Event:** samplingProtocol: Sweep net capture; eventDate: 2011-08-11; **Record Level:** collectionID: Tjo159; institutionCode: FCUL; collectionCode: Entomology_PCS
- **Type status:** Other material. **Occurrence:** recordedBy: Vera Nunes; individualCount: 1; sex: male; **Location:** country: Portugal; stateProvince: Algarve; verbatimLocality: Castro Marim; verbatimLatitude: 37°11'10.9"N; verbatimLongitude: 7°29'02.1"W; **Event:** samplingProtocol: Sweep net capture; Acoustic recording; eventDate: 2011-08-02; **Record Level:** collectionID: Tjo137; institutionCode: FCUL; collectionCode: Entomology_PCS
- **Type status:** Other material. **Occurrence:** recordedBy: Vera Nunes; individualCount: 1; sex: male; **Location:** country: España; stateProvince: Huelva; verbatimLocality: Cartaya; verbatimLatitude: 37°15'38.4"N; verbatimLongitude: 7°07'43.5"W; **Event:** samplingProtocol: Sweep net capture; Acoustic recording; eventDate: 2013-07-17; **Record Level:** collectionID: Tjo3557; institutionCode: FCUL; collectionCode: Entomology_PCS
- **Type status:** Other material. **Occurrence:** recordedBy: Vera Nunes; individualCount: 1; sex: male; **Location:** country: España; stateProvince: Huelva; verbatimLocality: Cartaya; verbatimLatitude: 37°15'38.4"N; verbatimLongitude: 7°07'43.5"W; **Event:** samplingProtocol: Sweep net capture; Acoustic recording; eventDate: 2013-07-18; **Record Level:** collectionID: Tjo3562; institutionCode: FCUL; collectionCode: Entomology_PCS
- **Type status:** Other material. **Occurrence:** recordedBy: Vera Nunes; individualCount: 1; sex: male; **Location:** country: España; stateProvince: Huelva; verbatimLocality: Cartaya; verbatimLatitude: 37°15'38.4"N; verbatimLongitude: 7°07'43.5"W; **Event:** samplingProtocol: Sweep net capture; Acoustic recording; eventDate: 2013-07-18; **Record Level:** collectionID: Tjo3566; institutionCode: FCUL; collectionCode: Entomology_PCS
- **Type status:** Other material. **Occurrence:** recordedBy: Vera Nunes; individualCount: 1; sex: male; **Location:** country: España; stateProvince: Huelva; verbatimLocality: Cartaya; verbatimLatitude: 37°14'03.7"N; verbatimLongitude: 7°03'56.8"W; **Event:** samplingProtocol: Sweep net capture; eventDate: 2013-07-18; **Record Level:** collectionID: Tjo3577; institutionCode: FCUL; collectionCode: Entomology_PCS

#### Description

##### Taxonomic identification

**Figure 2.**
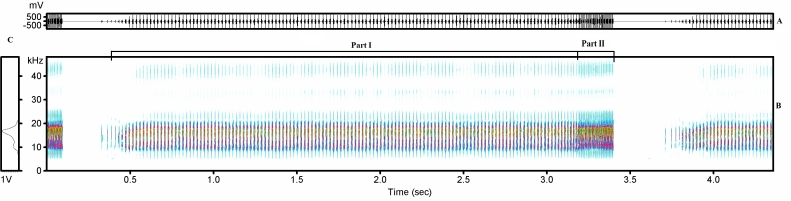
Calling song profile from a *Tettigettalna josei* male (ID 3562) recorded in Cartaya (Huelva, Spain). A – Oscillogram (amplitude vs. time), B – sonogram or spectrogram (frequency vs. time) and C – mean amplitude spectrum (frequency vs. amplitude).

Specimens collected in Spain were identified based on acoustic, morphological and genetic analysis. Acoustic analysis of the calling song of three males (Fig. 2, Table 1, Suppl. material 1) confirmed that their song’s profile is in agreement with previous descriptions of *Tettigettalna josei* acoustics (Fonseca 1991). The calling song is composed of the repetition of a long sequence of phrases. Each phrase includes two parts, Part I with a long sequence of echemes separated by very short intervals and Part II shorter than Part I, at the end of the phrase, with echemes produced continuously and ever decreasing inter-echeme interval duration. *Tettigettalna josei* specimens have a broad spectrum near 9 – 22.5 kHz with maximum energy around 17 kHz. For time domain variables, results obtained for Spanish specimens indicated an echeme duration ranging from 0.002 to 0.009s, with an average value of 0.004s. For the echeme period we found a range of 0.002 to 0.058s with an average of 0.015s. Morphological measurements of specimens collected in Spain are presented in Table 2. We found an average of 19.21 mm for total body length and 16.22 mm for hindwing length. These values are in general agreement with the ones previously reported for *Tettigettalna josei* (Boulard 1982). Sequences of cytochrome *c* oxidase I (COI) obtained from the three specimens collected in Spain (GenBank: KF977503–KF977505) were identical to the ones reported in Nunes et al. (2014) for *Tettigettalna josei* (GenBank: KC807267–KC807274) thus confirming species identification.

##### Genetic variation

We combined COI sequences from Nunes et al. (2014) and from this study in a dataset composed by 23 male specimens of *Tettigettalna josei* from several locations across its distribution (Table 4) and constructed a minimum spanning network (Fig. 5a). The dataset included eight variable sites, resulting in nine haplotypes that differ from each other by single mutations. All mutations result in synonymous changes in the protein. Haplotypes H1 and H6 were the ones found in higher frequencies, but they were detected only in a few sampled locations, indicating that the distribution of some haplotypes tends to be localized (Fig. 5b).

#### Distribution

**Figure 3.**
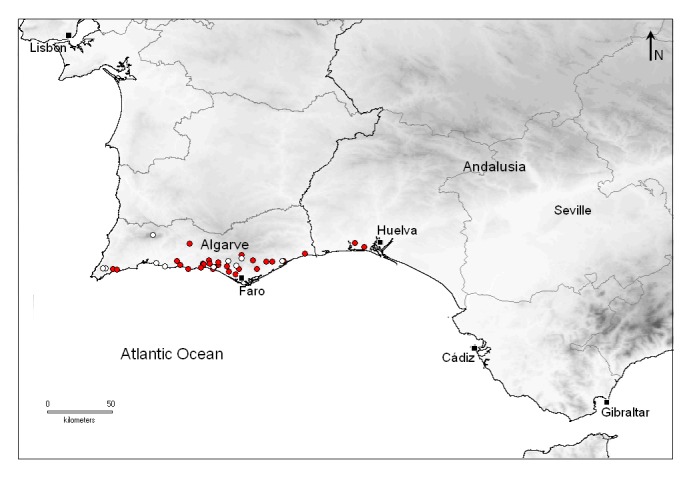
Map of occurrence of *Tettigettalna josei* in the south of the Iberian Peninsula, showing former documented populations in Algarve (Portugal) according to Sueur et al. (2004) (white circles) and populations recorded during our field surveys from 2011-2013 (red circles).

**Figure 5.**
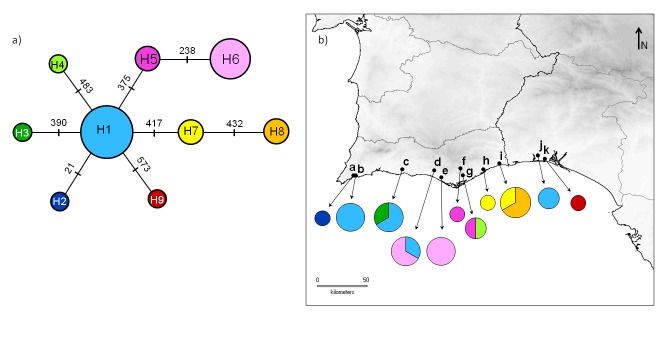
Minimum spanning network (a) for 23 sequences of cytochrome *c* oxidase I (COI) from males of *Tettigettalna josei* and the geographical distribution (b) of each haplotype. Numbers in the network correspond to the position of each mutation in the 581 base pairs sequences. Letters in the map for each sampled location are the same as in Table 4. Circle size is proportional to the number of specimens analysed (large = 3, medium = 2 and small = 1).

Calling songs from males of *Tettigettalna josei* were consistently heard in the region of Algarve, from the west Atlantic coast until the surroundings of the easternmost town, Vila Real de S. António (Fig. 3). A set of new locations and georeferenced coordinates of occurrence of *Tettigettalna josei* are listed in Table 3, but they should not be considered as an exhaustive list. *Tettigettalna josei* is quite widespread in Algarve, reaching high densities (> 10 singing males) in open habitats covered with low vegetation (small bushes and dry grass) and well exposed to sunlight (Fig. 4). Consequently, their numbers were low in cultivated tree groves where herbaceous vegetation was removed but were easily found in uncultivated fields or small patches of marginal vegetation by roads, in the periphery of villages and in secondary dunes and cliffs near the sea. *Tettigettalna josei* was found in sympatry with two other species belonging to the same genus, *Tettigettalna argentata* and *Tettigettalna mariae* (Table 3). Males of *Tettigettalna josei* were also found singing on trees, but they usually sing at heights below three meters, unlike their congenerics *Tettigettalna argentata* or *Tettigettalna mariae*, which often sing perched on high pine trees.

In July 2013, *Tettigettalna josei* was also found in small numbers in Cartaya (Huelva, Spain), which extends its known distribution to Spain. This same area was visited the year before (see Simões et al. 2013), but *Tettigettalna josei* was not found then. We surveyed other provinces of Andalusia in July of 2012 and 2013, but *Tettigettalna josei* was not found so far in any other areas besides Huelva.

#### Biology

##### Reproductive behaviour

**Figure 6.**
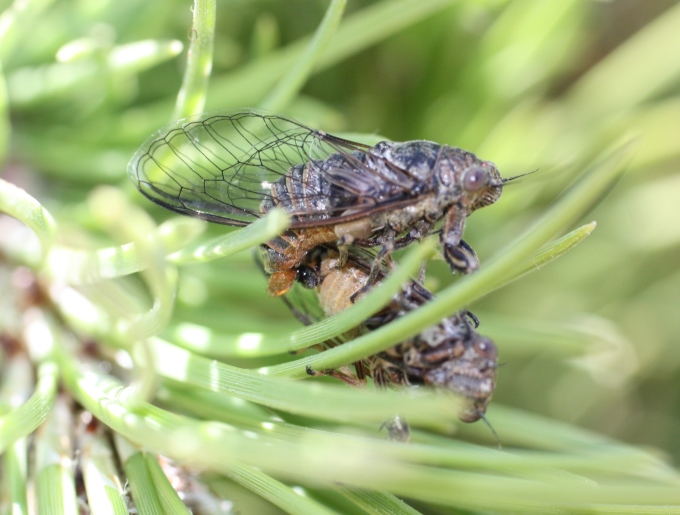
A couple of *Tettigettalna josei* during copulation observed in July 2013 near Sesmarias (37°04'38.6"N, 8°18'28.9"W), in Algarve, Portugal.

During our fieldwork we observed the copulatory mating behaviour of *Tettigettalna josei*. We had witnessed a few ongoing copulations in other *Tettigettalna* species (*Tettigettalna argentata* and *Tettigettalna helianthemi*) but had never seen how the process is initiated. One male of *Tettigettalna josei* was first noticed in a branch tip of a small stone pine (*Pinus pinea*) while singing and its unusual behaviour caught our attention. The male was moving frantically up and down or circling while singing. We noticed a female standing still in the same branch but on the opposite side of the male, thus out of sight of the male. The female was standing still and produced wing-flicks at regular and short intervals (see Boulard (2006) for a review on cicadas wing-flicking). After a few wing-flicks the male eventually moved into the females’ direction, turned around the branch and made body contact with her. The male immediately mounted on the female’s dorsum and initiated a sideway copulation (Fig. 6). The couple stood quietly and in silence for about three minutes, being the female’s front legs firmly attached to the branch. When the couple become apart, the female remained in the branch while the male took off a minute later to a nearby branch and resumed his calling song. A female of this species was seen laying their eggs on a Fennel stem (*Foeniculum vulgare*) at about 1 m from the soil. Cicadas of genus *Tettigettalna* are particularly vulnerable during copulation and oviposition because they tend to resist fleeing away if threatened.

#### Taxon discussion

The present data clarify the currently known distribution range of *Tettigettalna josei* in the south of the Iberian Peninsula, encompassing the region of Algarve (Portugal) and the province of Huelva (Spain). Morphological, acoustic and genetic analyses confirmed the identity of specimens collected in Cartaya (Huelva) as belonging to *Tettigettalna josei*.

Previous records on the distribution of this small cicada species were sparse and limited to Portugal (Sueur et al. 2004). Hence the species has been considered until now as endemic to Portugal. The new record of *Tettigettalna josei* in Spain in July 2013 refutes this status and the species must now be added to the list of the Spanish cicadas and, therefore, should be considered as an Iberian endemism.

The region of Huelva was previously surveyed in August 2012 with the detection of *Tettigettalna mariae* (Simões et al. 2013) but not of *Tettigettalna josei*. This could be explained by the low densities of *Tettigettalna josei* found in Spain, since only about five singing males were detected in the region in 2013. Additionally, we have noted that the emergence peak of *Tettigettalna josei* might be slightly earlier (June-July) as compared to other *Tettigettalna* species (*Tettigettalna argentata* and *Tettigettalna mariae*: mostly July). Consequently, the number of singing males of *Tettigettalna josei* should decline earlier, in mid-August, justifying why we might have missed *Tettigettalna josei* in Spain during our surveys in mid-August 2012.

Data obtained so far indicates that populations of *Tettigettalna josei* are acoustic, morphological and genetically homogeneous throughout the distribution range of the species. The genetic analysis of COI gene showed no evidence of population structure. Haplotypes differ by single mutations from each other and form a star-like haplotype network. In spite of this, some differences in the distribution and frequency of each COI haplotype seem to reflect the expected trend for low dispersal in these cicadas. As demonstrated before in a species of genus *Cicada* (Simões and Quartau 2007), emerged specimens usually experience limited dispersal during the mating season, remaining near the emergence site, where the singing males may form choruses.

The distribution of *Tettigettalna josei* overlaps with an area under severe human pressure. The coastline of Algarve has been intensively exploited for beach tourism and golf. However, land management associated with tourism facilities seem to have less impact on the persistence of *Tettigettalna josei* than the perturbation caused by farming practices that deplete the shrub and grass cover of the soil (e.g. plowing, harvesting or intensive grazing). Fortunately for cicadas, these practices are not severely intense in the coastal region of Algarve and crops are usually small sized and patchy, allowing populations of *Tettigettalna josei* to persist all over the region. In contrast, monocultures such as olive and stone pine woods in the Spanish region of Andalusia occupy extensive areas and are regularly maintained to restrain the growth of shrub-like vegetation under the trees. These differences in land management might help to explain the small effective numbers of *Tettigettalna josei* detected so far in Spain, and favours instead the prevalence of *Tettigettalna* species that are frequently found on trees, such as *Tettigettalna mariae* or *Tettigettalna aneabi*.

## Supplementary Material

Supplementary material 1 Calling song from a Tettigettalna josei male (ID 3562) recorded in Cartaya (Huelva, Spain). Data type: Audio recording
File: oo_5460.m4a Vera Nunes and Raquel Mendes

XML Treatment for Tettigettalna josei

## Figures and Tables

**Figure 4a.**
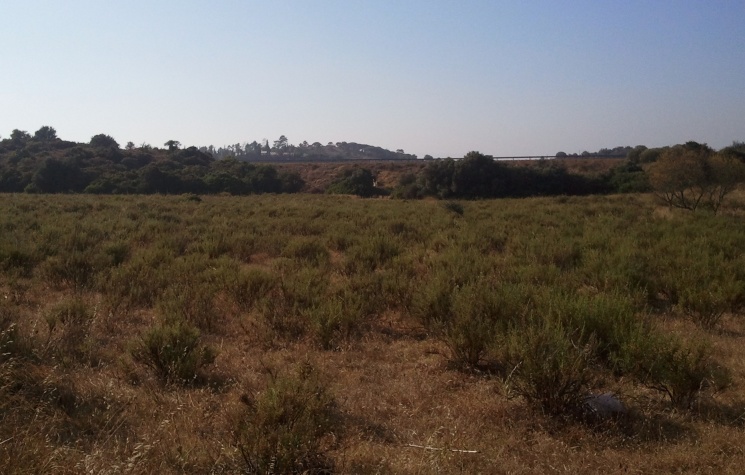


**Figure 4b.**
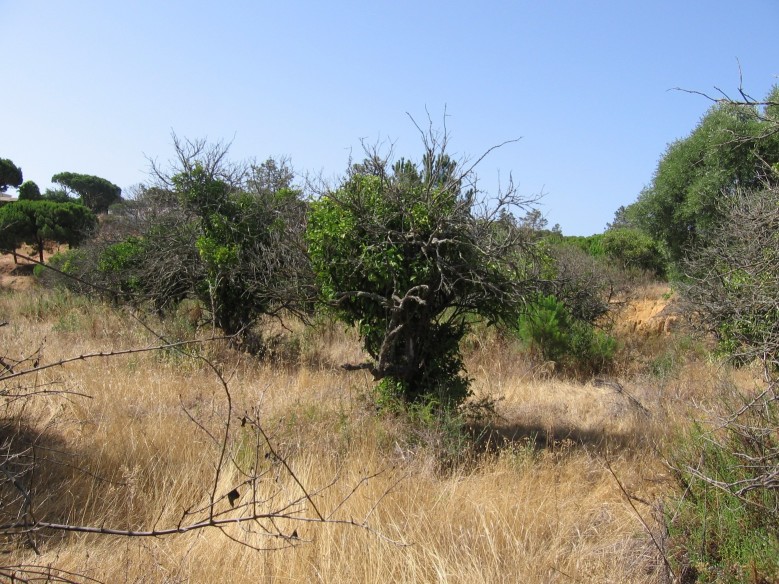


**Figure 4c.**
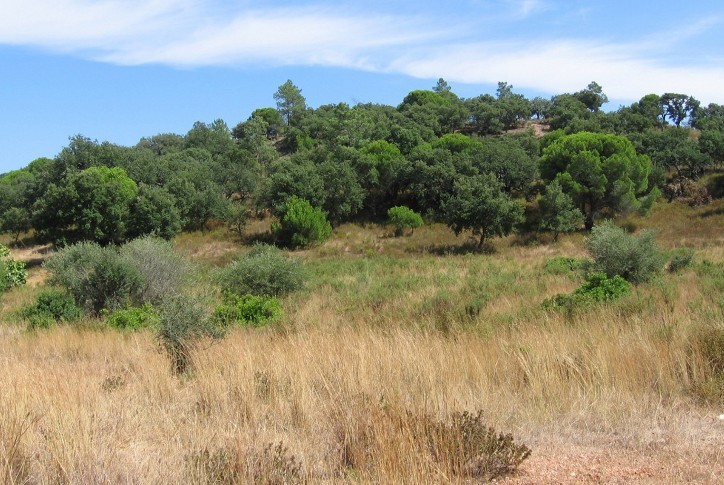


**Table 1.** Descriptive statistics of the acoustic variables from two males (ID 3562, 3566) of *Tettigettalna josei* collected in Cartaya (Huelva, Andalusia). Time variables are given in seconds and frequency variables in kHz.

	Ech/s	Echeme duration (s)	Echeme period (s)	Inter-echeme interval (s)	Peak frequency	Minimum frequency	Maximum frequency
Average	45.50	0.004	0.015	0.021	16.67	8.87	20.46
Maximum	46.35	0.009	0.058	0.077	18.30	9.70	22.50
Minimum	44.65	0.002	0.002	0.005	14.60	4.10	18.00

**Table 2.** Morphometric values (in mm) for each *Tettigettalna josei* male captured in Cartaya (Huelva, Andalusia): TL – total length, BL – body length, WS – wingspan, aWL – right hindwing length, HW – head width, MW – mesonotum width and ACD – width at the level of the auditory capsules.

Specimen ID	TL	BL	WS	aWL	HW	MW	ACD
3557	18.46	13.65	33.93	15.73	4.36	4.03	4.42
3562	20.41	15.60	36.66	17.16	4.55	4.49	4.49
3566	18.59	13.65	35.23	15.73	4.23	4.10	4.16
3577	19.37	14.95	32.50	16.25	4.49	4.36	4.42
Average	19.21	14.46	34.58	16.22	4.41	4.25	4.37

**Table 3.** List of localities and GPS coordinates in the south of the Iberian Peninsula where specimens of *Tettigettalna josei* were detected. Type of observation: Ao – Audio only (sound heard but not recorded), Ar – Audio recording and C – Captured.

Locality	Region	Country	GPS coordinates (degrees minutes seconds)	Date	Type of observation	Sympatry with other *Tettigettalna* species
Budens	Algarve	Portugal	37°04'22.9"N, 8°48'43.9"W	27/07/2011	C	
Budens	Algarve	Portugal	37°04'45.2"N, 8°50'11.6"W	27/07/2011	C	*Tettigettalna argentata*
Porches	Algarve	Portugal	37°08'09.4"N, 8°23'04.2"W	26/07/2011	Ar, C	*Tettigettalna argentata*
Armação de Pêra	Algarve	Portugal	37°06'23.4"N, 8°21'47.1"W	22/06/2013	Ao	
Sesmarias	Algarve	Portugal	37°04'38.6"N, 8°18'28.9"W	26/07/2013	Ao	
S. Bartolomeu de Messines	Algarve	Portugal	37°15'25.7"N, 8°17'55.6"W	28/07/2011	Ao	*Tettigettalna argentata*
Monte Choro	Algarve	Portugal	37°05'18.8"N, 8°12'58.4"W	10/08/2012	Ao	
Vale Navio	Algarve	Portugal	37°06'43.2"N, 8°12'09.0"W	26/06/2012	Ao	*Tettigettalna mariae*
Vale Navio	Algarve	Portugal	37°06'34.6"N, 8°12'07.8"W	26/06/2012	Ao	*Tettigettalna mariae*
Vale Navio	Algarve	Portugal	37°07'05.4"N, 8°12'02.3"W	09/08/2012	Ao	*Tettigettalna argentata*
Vale Navio	Algarve	Portugal	37°06'37.1"N, 8°12'00.2"W	26/06/2012	Ao	
Boliqueime	Algarve	Portugal	37°08'23.9"N, 8°09'41.0"W	29/07/2011	Ao	*Tettigettalna argentata*
Boliqueime	Algarve	Portugal	37°07'01.5"N, 8°09'16.8"W	29/07/2011	Ao	
Praia da Falésia	Algarve	Portugal	37°04'36.5"N, 8°08'00.6"W	25/06/2012	Ao	
Vilamoura	Algarve	Portugal	37°05'27.3"N, 8°07'27.5"W	25/06/2012	Ao	
Vale Judeu	Algarve	Portugal	37°06'21.2"N, 8°05'42.8"W	26/06/2012	Ao	*Tettigettalna mariae*
Vale Judeu	Algarve	Portugal	37°07'39.8"N, 8°05'36.1"W	12/07/2011	Ar, C	
Almancil	Algarve	Portugal	37°05'47.7"N, 8°01'52.3"W	07/08/2012	Ao	
Quinta do Lago	Algarve	Portugal	37°03'35.2"N, 8°01'16.3"W	01/08/2012	Ar, C	*Tettigettalna mariae* and *Tettigettalna argentata*
Faro	Algarve	Portugal	37°02'29.1"N, 7°58'18.1"W	07/08/2012	Ao	*Tettigettalna mariae*
Santa Bárbara de Nexe	Algarve	Portugal	37°04'48.8"N, 7°56'55.4"W	08/08/2012	Ao	
S. Brás de Alportel	Algarve	Portugal	37°10'33.5"N, 7°55'52.8"W	04/08/2011	Ao	*Tettigettalna argentata*
S. Brás de Alportel	Algarve	Portugal	37°08'14.8"N, 7°50'52.4"W	04/08/2011	Ar, C	*Tettigettalna argentata*
Moncarapacho	Algarve	Portugal	37°04'41.3"N, 7°49'16.6"W	03/08/2011	Ar, C	*Tettigettalna argentata*
Estiramantens	Algarve	Portugal	37°07'49.2"N, 7°45'15.6"W	03/08/2011	Ao	*Tettigettalna argentata*
Santo Estevão	Algarve	Portugal	37°07'46.3"N, 7°42'53.6"W	03/08/2011	Ao	
Tavira	Algarve	Portugal	37°08'02.0"N, 7°38'04.2"W	11/08/2011	C	
Castro Marim	Algarve	Portugal	37°11'10.9"N, 7°29'02.1"W	02/08/2011	Ar, C	*Tettigettalna argentata*
Cartaya	Huelva	Spain	37°15'38.4"N, 7°07'43.5"W	18/07/2013	Ar, C	*Tettigettalna mariae*
Cartaya	Huelva	Spain	37°14'03.7"N, 7°03'56.8"W	18/07/2013	C	*Tettigettalna mariae*

**Table 4.** List of males of *Tettigettalna josei* sequenced for the mitochondrial gene cytochrome *c* oxidase I (COI).

Specimen	COI haplotype	NCBI Ac. n.	Source	Location	Location code	Latitude	Longitude
Tjo119	H2	KF977491	This study	Budens	a	37°04'45.2"N	8°50'11.6"W
Tjo120	H1	KC807267	Nunes et al. 2014	Budens	b	37°04'22.9"N	8°48'43.9"W
Tjo121	H1	KC807268	Nunes et al. 2014	Budens	b	37°04'22.9"N	8°48'43.9"W
Tjo122	H1	KF977492	This study	Budens	b	37°04'22.9"N	8°48'43.9"W
Tjo106	H3	KC807272	Nunes et al. 2014	Porches	c	37°08'09.4"N	8°23'04.2"W
Tjo113	H1	KF977493	This study	Porches	c	37°08'09.4"N	8°23'04.2"W
Tjo116	H1	KC807271	Nunes et al. 2014	Porches	c	37°08'09.4"N	8°23'04.2"W
Tjo58	H1	KC807273	Nunes et al. 2014	Vale Judeu	d	37°07'39.8"N	8°05'36.1"W
Tjo64	H6	KC807274	Nunes et al. 2014	Vale Judeu	d	37°07'39.8"N	8°05'36.1"W
Tjo66	H6	KF977494	This study	Vale Judeu	d	37°07'39.8"N	8°05'36.1"W
Tjo309	H6	KF977495	This study	Quinta do Lago	e	37°03'35.2"N	8°01'16.3"W
Tjo355	H6	KF977496	This study	Quinta do Lago	e	37°03'35.2"N	8°01'16.3"W
Tjo362	H6	KF977497	This study	Quinta do Lago	e	37°03'35.2"N	8°01'16.3"W
Tjo145	H5	KF977498	This study	S. Brás de Alportel	f	37°08'14.8"N	7°50'52.4"W
Tjo141	H5	KF977499	This study	Moncarapacho	g	37°04'41.3"N	7°49'16.6"W
Tjo154	H4	KF977500	This study	Moncarapacho	g	37°04'41.3"N	7°49'16.6"W
Tjo159	H7	KF977501	This study	Tavira	h	37°08'02.0"N	7°38'04.2"W
Tjo135	H7	KC807270	Nunes et al. 2014	Castro Marim	i	37°11'10.9"N	7°29'02.1"W
Tjo137	H8	KF977502	This study	Castro Marim	i	37°11'10.9"N	7°29'02.1"W
Tjo140	H8	KC807269	Nunes et al. 2014	Castro Marim	i	37°11'10.9"N	7°29'02.1"W
Tjo3557	H1	KF977503	This study	Cartaya	j	37°15'38.4"N	7°07'43.5"W
Tjo3562	H1	KF977504	This study	Cartaya	j	37°15'38.4"N	7°07'43.5"W
Tjo3577	H9	KF977505	This study	Cartaya	k	37°14'03.7"N	7°03'56.8"W
